# Implication of a Key Region of Six *Bacillus cereus* Genes Involved in Siroheme Synthesis, Nitrite Reductase Production and Iron Cluster Repair in the Bacterial Response to Nitric Oxide Stress

**DOI:** 10.3390/ijms22105079

**Published:** 2021-05-11

**Authors:** Constance Porrini, Cyprien Guérin, Seav-Ly Tran, Rozenn Dervyn, Pierre Nicolas, Nalini Ramarao

**Affiliations:** 1 Micalis Institute, AgroParisTech, INRAE, Université Paris-Saclay, 78350 Jouy-en-Josas, France; constanceporrini@gmail.com (C.P.); seav-ly.tran@inrae.fr (S.-L.T.); rozenn.dervyn@inrae.fr (R.D.); 2 MaIAGE, INRAE, Université Paris-Saclay, 78350 Jouy-en-Josas, France; cyprien.guerin@inrae.fr (C.G.); pierre.nicolas@inrae.fr (P.N.)

**Keywords:** *Bacillus cereus*, nitric oxide, iron cluster repair, transcriptomic

## Abstract

Bacterial response to nitric oxide (NO) is of major importance for bacterial survival. NO stress is a main actor of the eukaryotic immune response and several pathogenic bacteria have developed means for detoxification and repair of the damages caused by NO. However, bacterial mechanisms of NO resistance by Gram-positive bacteria are poorly described. In the opportunistic foodborne pathogen *Bacillus cereus*, genome sequence analyses did not identify homologs to known NO reductases and transcriptional regulators, such as NsrR, which orchestrate the response to NO of other pathogenic or non-pathogenic bacteria. Using a transcriptomic approach, we investigated the adaptation of *B. cereus* to NO stress. A cluster of 6 genes was identified to be strongly up-regulated in the early phase of the response. This cluster contains an iron-sulfur cluster repair enzyme, a nitrite reductase and three enzymes involved in siroheme biosynthesis. The expression pattern and close genetic localization suggest a functional link between these genes, which may play a pivotal role in the resistance of *B. cereus* to NO stress during infection.

## 1. Introduction

Nitric oxide (NO) stress is an important component of the eukaryotic immune response. NO is a free radical, which can be a powerful oxidizing and nitrating agent, and can react with many components of the bacterial cell [1]. In particular, it can bind and modify the properties of nucleic acids, lipids, sugars and proteins. NO is also able to bind to transition metal, such as Fe, Mn, Cu, etc., present in metalloproteins, modifying their activity. All these modifications can lead to bacterial cell death.

Pathogenic bacteria have developed several mechanisms to defend themselves against this antimicrobial weapon [2], although the entire machinery triggered by bacteria is not fully understood nowadays. In bacteria such as *Escherichia coli* or *Staphylococcus aureus*, the bacterial response to NO occurs in four steps: sensing, signaling, detoxification and repair [3,4]. When bacteria detect NO with regulators (i.e., norR) or NO-sensitive transcriptional factors, such as NsrR, regulation pathways are induced, leading to expression of genes involved in the resistance to NO. Detoxification proteins, such as NOR and Hmp, transform NO in the cytosol into less toxic compounds like nitrate or nitrous oxide. Damage repair proteins, such as Mfd, YtfE and ScdA, restore the integrity of nucleic acid and proteins, respectively [3,4,5,6].

YtfE and ScdA belongs to the RIC family (iron cluster repair). They are involved in repairing iron-sulfur clusters, which are groups of iron and sulfur atoms present on certain metalloproteins. NO can act on these groups by nitrosylating or by disassembling them, modifying the activity of the enzymes. In *E. coli,* YtfE restores the activity of iron cluster enzymes and its transcription is regulated by NsrR, FNR and Fur [3,7]. While NsrR is a repressor of the expression of NO response genes, FNR is an activator of the expression of genes encoding the nitrate, nitrite, fumarate reductase system [8]. Both, NsrR and FNR have an iron-sulfur cluster that is disassembled by NO, which affects their DNA binding [9]. Fur is an iron-responsive transcription factor uptake regulator controlling the iron homeostatic response [10].

*Bacillus cereus* is a spore forming human pathogen and is currently the second leading cause of collective foodborne outbreaks in France after *Staphylococcus aureus* [11,12,13]. *B. cereus* can also cause severe systemic infection [14,15] especially in newborn children [16,17]. During infection, *B. cereus* is able to resist the host immune system, surviving phagocytosis by macrophages [18,19] and inducing their apoptosis [20,21]. Macrophage major mechanisms of cytotoxicity is the massive production of NO, and *B. cereus* is particularly resistant to NO through the implication of the Mfd protein during DNA repair [5,6,22]. Nevertheless, *B. cereus* does not seem to carry most of the common genes involved in NO response, such as *nor*, *nsrR*, *norR* and the genetic basis of its NO response is unknown.

In this study, we investigate NO defense mechanisms of this emerging pathogen by a transcriptomic approach. We identified a cluster of six genes, all up-regulated, in the early response to NO stress. They encode a RIC enzyme, a nitrite reductase and a siroheme biosynthesis enzyme. Their expression pattern and close genetic localization suggest a functional link between these genes during *B. cereus* fight against NO stress.

## 2. Material and Method

### 2.1. Bacterial Strains

The strain used for experiments is *Bacillus thuringiensis* 407 Cryˉ also referred to as Bc407. It is an acrystalliferous strain cured of its *cry* plasmid [23], which is genetically close to the *Bacillus cereus* reference strain ATCC 14579 [24].

Genomes used for sequence alignments are:

*Bacillus thuringiensis* Bc407: NC_018877.1

*Bacillus cereus* ATCC 14579: NC_004722.1

*Bacillus anthracis* CZC5 DNA: AP018443.1

*Bacillus subtilis* subsp. *subtilis* str. ATCC 6051: NZ_CM000488.1

*Staphylococcus aureus subsp. aureus* taxid:46170

### 2.2. Growth and Stress Conditions

Bc407 was grown in LB medium at 37 °C with agitation (200 rpm) until the end of the exponential growth phase (OD_600_ = 2). Then, bacteria were diluted 1/100 in RPMI 1640 Medium GlutaMAX^TM^ (SKU 61870-010, Thermofisher), and exposed to diverse stresses for 15 min to 60 min at 37 °C without agitation.

NOC5 (146724-82-5, Calbiochem) was used as NO donor at a final concentration of 10 and 50 µM. Sodium nitrite (7632-00-0 Sigma-Aldrich) was used at 2.5 mM, Potassium nitrate (7757-79-1, Merck) at 20 mM and hydrogen peroxide solution (7722-84-1, Sigma-Aldrich) at 0.03% (*V*/*V*). The quantity of nitrate in solution was measured using the Griess Reagent System (G2930, Promega) following the manufacturer protocol.

Bacteria were starved from iron during incubation in iron-free RMPI medium for one hour at 37 °C with agitation. Then iron stresses (excess or starvation) were induced during 15 min with Iron III citrate (6100-05-6, Sigma-Aldrich) at 81 µM, and 2,2′-Bipyridyl (366-18-7, Sigma-Aldrich) at 4,4 μM as an iron chelator, respectively [25,26].

### 2.3. RNAseq Library Preparation and Data Analysis

Following stresses, bacterial pellets were frozen in liquid nitrogen and introduced in Lysing Matrix B tubes (MP Biomedicals) and high-speed homogenized in a FastPrep-24™ 5G Instrument in presence of phenol. Impurities were extracted from the aqueous phase in three steps: one step with trizol and two with chloroform. The RNAs in the aqueous phase were then precipitated with isopropanol and dissolved in purified water [27]. The quality of the RNA samples was tested by bioanalyzer (Agilent) [28]. Only samples with a RIN (RNA integrity number) above 7 were used for experiments.

RNA samples of Bc407 were prepared as described above with different levels NO stress (0, 10 and 50 µM of NO) at different time points (0, 15 and 60 min after NO stress) as described in Supplementary Table (Table S1). Transcriptome library preparation and sequencing was performed by I2BC Paris-Saclay platform on the Illumina NextSeq sequencer to generate paired-end 40 bp reads bearing strand specificity. Reads were trimmed based on sequencing quality using Sickle (v1.33) [29] and mapped on Bc407 reference genome assembly (NC_018877.1) using Bowtie2 (2.2.6; options “-N 1-L 16-R 4”) [30] before read-count aggregation on the sense and antisense strand of each transcribed region with Htseq-count (0.10.0; standard options). Experiments were made in triplicates to allow statistical differential expression analysis. RPKM (Reads Per Kilobase Million) normalization [31] served for a first level of exploratory analysis. Differential expression analysis of Bc407 relied on R library “DESeq2” [32] and associated “median ratio method” normalization procedure. DESeq2 *p*-values were converted into q-values using R library “fdrtool” [33]. The genes were considered as up-regulated or down-regulated when q-value ≤ 0.05 and log2 fold change ≥ 1 or ≤−1, respectively. Raw transcriptomic data and differential expression analysis are accessible through GEO Series accession number GSE168681 (https://www.ncbi.nlm.nih.gov/geo/query/acc.cgi?acc=GSE168681, accessed on 1 June 2021).

### 2.4. Bio-Informatic Analysis of the Six Genes Region

The region of interest was defined between the nucleotides 2,097,000 and 2,140,000 (NC_018877.1) for all bioinformatical analysis. The mapped paired-end reads were visualized along the genome with IGV tools [34]. The prokaryotic promoter search was performed with the Fruitfly seq tool (https://www.fruitfly.org/seq_tools/promoter.html, accessed on 1 June 2021), and only results with a score between 0.8 and 1 were considered. The bacterial operon and gene prediction were searched with the FGENESB tool [35]. The presence of putative regulator-binding regions was assessed by scanning for matches to consensus sequences listed in Table 1 using the web version of FIMO 5.0.3. Only sequence matches with a *p*-value < 10^−6^ and q-value < 0.01 were taken into account [36]. The Fur box was identified using the pairwise sequence alignment with EMBOSS Needle.

### 2.5. Reverse Transcription Quantitative PCR (RT-qPCR)

RT-qPCR was performed to analyze gene expression under different stress conditions. The RNA harvested after stresses were reverse transcribed with Transcriptor First Strand cDNA Synthesis Kit (04897030001, Roche) followed by the PCR reaction with Brilliant III Ultra-fast SYBR Green qPCR Master Mix (600828, Agilent) in the StepOne^TM^ Real-Time PCR system (Applied Biosystems) using the primer pairs in Table 2. The experiments were performed according to the following: 1 cycle at 95 °C of 3 min and 40 cycles at 95 °C for 5 s and at 60 °C for 10 s. Each sample was quantified in technical duplicates.

The gene *rpoA* (BTB_RS00795) was used as endogenous control, and was included for each sample. The condition without stress was used as the reference condition. The average and the standard deviations of RQ for each condition were calculated from the expression values of biological triplicates. As a result, the relative quantification of gene expression was shown. RQ = Relative quantification = 2^−ΔΔCt^

## 3. Results

### 3.1. B. cereus Transcriptomic Response to NO Stress

**Figure 1:**
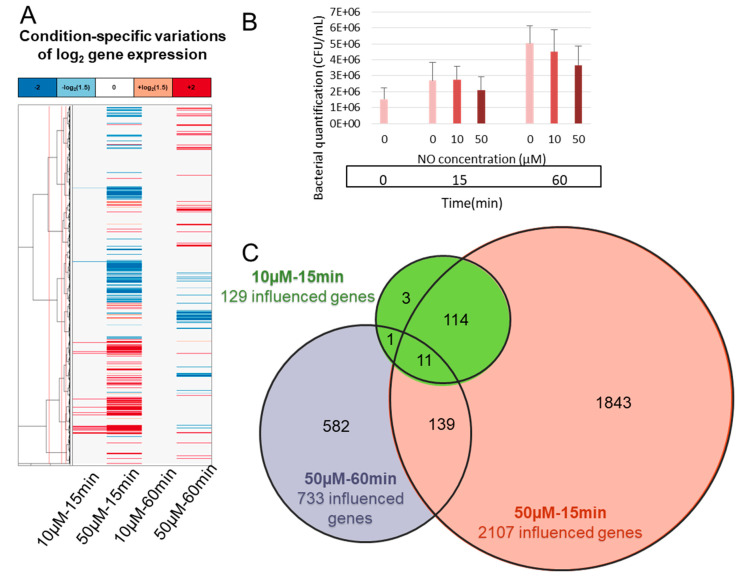
RNA sequencing on *Bacillus cereus* after nitric oxide stress. Bacteria were exposed to NO stress (10 µM or 50 µM) for 15 or 60 min. mRNA was extracted and sequenced by NextSeq. (**A**) Differentially expressed genes profiles based on reads mapped on reference genome of Bc407 (NC_018877.1) aggregated at gene level shows the overexpressed (in red) or under expressed (in blue) genes. Genes are clustered in co-expression groups; (**B**) bar chart representing the bacterial load for each condition; (**C**) Venn diagram showing the size and overlap of the sets of genes up- or down-regulated in each condition (empty set for the condition 10 µM-60 min).

NO stress was applied to the bacteria for either a short time (15 min) or a longer time (60 min) at two different concentrations (10 and 50 µM), and *B. cereus* response was investigated (Figure 1). The actual NO concentration was verified by the Griess assay for each condition. The average concentrations were close to those expected (9.4 and 60.1 µM) after 15 min and they decreased slightly after 1 h (4.6 and 39.0 µM). The quantification of viable bacteria before and after the NO stress indicated only a slight impact of these NO stress conditions on bacterial survival.

The impact on gene expression was then investigated by a transcriptomic approach. The effect of NO on gene expression was higher at 15 min and dose dependent (Figure 1A,C). The number of significantly impacted (up- or down-regulated) genes after 15 min were 129 and 2107, respectively for 10 and 50 µM of NO, with 2107 genes representing approximately one third (2107/6758) of the entire *B. cereus* genome. However, after 60 min the number of up- or down-regulated genes dropped to 0 for 10 µM of NO and 733 for 50 µM of NO. This demonstrates a strong dose-dependent response, with approximately 16 times more genes impacted for 50 μM than for 10 µM, at 15 min.

**Figure 2:**
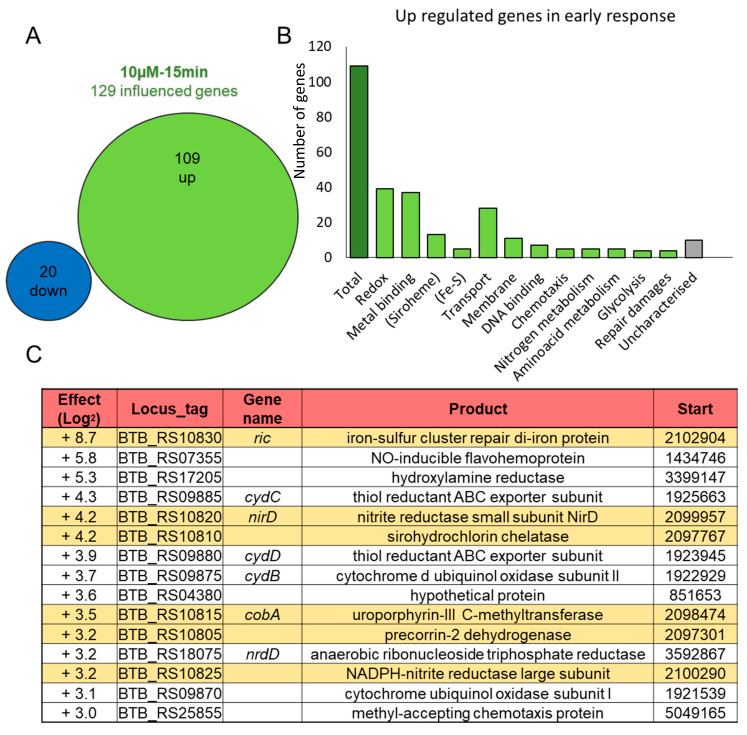
Characterization of the early response to NO stress by analysis of gene expression after 15 min and 10 µM of NO; (**A**) Venn diagram of the 129 genes found as impacted by the addition of 10 µM of NO; (**B**) Distribution of the 109 up-regulated genes in functional groups; (**C**) Listing of the 15 most impacted genes, all are up-regulated. The first column is the log2 fold change of the expression level. The six genes highlighted in yellow are in the same chromosomal region.

At 15 min, 97% of the genes identified as impacted by 10 μM NO were included in the set of genes impacted by 50 μM NO, (the four exceptions being BTB_RS05820, BTB_RS16655, BTB_RS18840, BTB_RS34095). By contrast, the overlap between the response at 15 min and 60 min was modest, with only 21% of the genes identified as impacted at 60 min and 50 μM NO already impacted at 15 min (Figure 1C). This indicates distinct phases in the transcriptomic response. We then focused on the early response to 10 µM NO, which was the most specific, with only 109 up- and 20 down-regulated genes (Figure 2, Supplementary Table S2).

The analysis of the genes up-regulated in these conditions identified one third as involved in redox process and another third in metal binding (Figure 2B), with 13 genes, which function is related to siroheme and 5 to iron-sulfur cluster. Figure 2C presents the 15 most impacted genes, all are highly up-regulated (from 8 to 400 times). During this early response, the most up-regulated gene is *ric,* which encodes an enzyme involved in the repair of iron-sulfur clusters.

### 3.2. A Chromosomal Region of 6 Genes over Activated during Early Response to NO Stress

**Figure 3:**
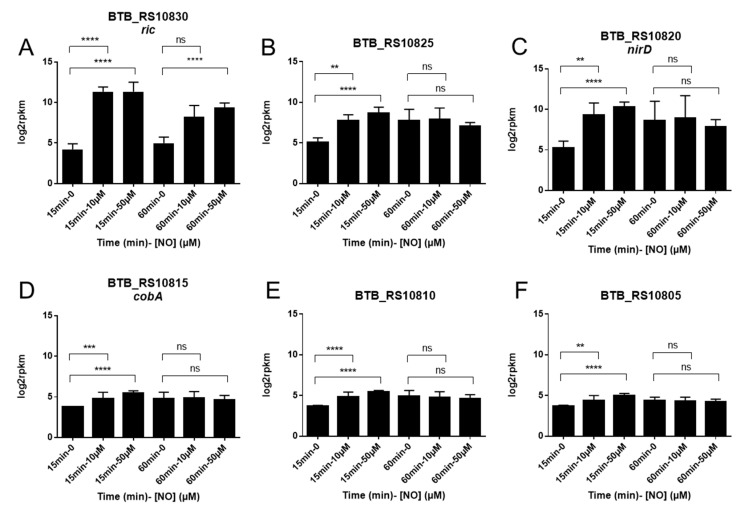
Expression of the 6 genes of the BTB_RS10830-BTB_RS10805 region as measured by RNAseq across time points and levels of NO stress. Gene expression level is counted in reads per kilobase of transcript, per million of mapped reads (represented here in log2-scale). Average and standard deviation from three independent experiments are shown. (Mann-Whitney, ns, not significant, q-value ** <0.01 *** <0.001, **** <0.0001); (**A**) BTB_RS10830 encoding RIC; (**B**) BTB_RS10825 encoding the large sub unit of the nitrite reductase; (**C**) BTB_RS10820, encoding the small sub unit of the nitrite reductase (NirD); (**D**) BTB_RS10815, encoding the uroporphyrin-III C-methyltransferase; (**E**) BTB_RS10810, encoding the sirohydrochlorin chelatase; (**F**) BTB_RS10805, encoding the precorrin-2 dehydrogenase.

**Figure 4:**
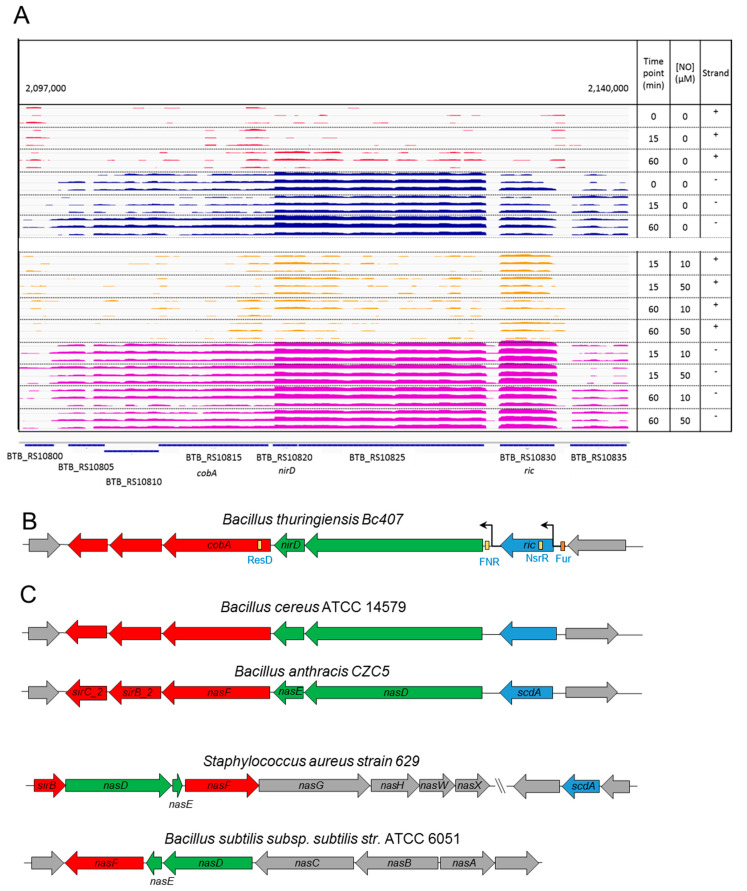
Organization and expression of the 6 genes of the BTB_RS10830-BTB_RS10805 region. (**A**) Quantification of transcription along the region. Representation of the number of pairs of reads (mRNA fragments) overlapping each position of the positive strand (red and yellow) and negative strand (blue and purple). Each colored area corresponds to one replicate in one condition: without NO (red and blue) or with NO (yellow and purple). The last line is the gene annotation of the reference strain *Bacillus cereus* Bc407 (NC_018877.1). (**B**) Schematic representation of the organization of the region in Bc407 chromosome (not to scale). The yellow boxes indicate the putative presence of regulatory binding regions. The black arrows indicate putative promoters. (**C**) Organization of the region in other *Firmicutes* species. The genes in grey do not belong to the 6 genes of the BTB_RS10830-BTB_RS10805 region.

Among the 15 most up-regulated genes at 15 min of the 10 µM NO stress, a chromosomal region of 6 genes, including *ric*, appears of utmost interest. Unsurprisingly, these six genes were also significantly up-regulated at 15 min of the 50 μM NO (Figure 3). The six genes are adjacent and co-directionally transcribed on the Bc407 chromosome, from locus tag BTB_RS10830 (*ric*) to BTB_RS10805 (Figure 4). They are encoding a RIC family protein, the large and small subunits of a nitrite reductase (BTB_RS10825 and BTB_RS10820), and three enzymes of the siroheme biosynthesis pathway: a uroporphyrin-III C-methyltransferase, a sirohydrochlorin chelatase, and a precorrin-2 dehydrogenase (BTB_RS10815, BTB_RS10810, BTB_RS10805). These genes are mainly involved in the early response to NO since after 60 min of NO stress, only the *ric* gene is still up-regulated (Figure 3).

In order to characterize this region, the IGV tool was used to visualize the mapping of sequenced transcripts along the region. The mapping occurs mostly in the negative strand (blue and purple) in agreement with the genome annotation (Figure 4A). The induction of the expression or the *ric* gene in presence of NO is clearly visible in this representation, as well as a gap of expression at the end of the gene, suggesting the presence of a transcription terminator after *ric*. Then the two subunits of the nitrite reductase seem to be co-transcribed at a same level and transcription drops substantially after these genes suggesting partial termination or RNA degradation. The three following genes involved encoding enzymes for the siroheme biosynthesis seem co-transcribed at a same level in a given sample. In view of these levels of expression, three sub-groups of genes can be identified within this region. The first contains only *ric* (in blue), then the two subunits of nitrite reductase (in green) and finally the genes of the siroheme biosynthesis (respectively, represented in blue, green and red in Figure 4B,C). To identify potential promoters, a sequence pattern analysis was performed with Fruitfly and FGENESB tools. Both programs predicted only two promoters, one upstream of the *ric* gene and the other upstream of the gene encoding the large subunit of the nitrite reductase (BTB_RS10825).

FIMO tool was used to identify putative binding sites for relevant transcription factors with known consensus motifs (Table 1). Results are shown in Figure 4B. Upstream of the *ric* gene a putative region of attachment of the NO-sensitive inhibitor, NsrR was identified. There is no homolog of *nsrR* in the Bc407 genome. But a PSI-Blast analysis highlighted three proteins (WP_000877649.1, WP_000704116.1, WP_001083465.1) with around 30% identity with *B. subtilis* NsrR (not shown). These proteins are of unknown functions, but they belong to the same Rrf2 family of transcriptional regulator as NsrR. Upstream of the BTB_RS10825 gene, a potential FNR binding site was identified and a putative ResD site is located in the first part of *cobA* gene (BTB_RS10815). Upstream of the *ric* gene, a putative Fur box was also identified by pairwise alignment, but its score was not high enough to be found with FIMO.

To assess the conservation of this region within the *B. cereus* group, other closely related bacteria were analyzed (Figure 4C). Homolog genes were found in other species and also co-localized in a same region of the chromosome. We confirmed that the region of six genes has a similar organization in *Bacillus cereus* ATCC 14579 than in Bc407. Moreover, the organization of this region is conserved in *Bacillus anthracis,* which also belongs to the *Bacillus cereus* group. However, this organization is not perfectly conserved in more distant species (Figure 4C). In *Staphylococcus aureus,* the homolog of *ric* is not in the neighborhood of the nitrite reductase genes. In *Bacillus subtilis,* the nitrite reductase and the siroheme biosynthesis genes were clustered, no *ric* homolog was found. Other more distant species were also examined (up to *Escherichia coli*, *Pseudomonas aeruginosa*, and *Mycobacterium tuberculosis)* but no region containing the associated three parts could be identified (results not shown). These data suggest that the organization of the region of these six genes is specific to a subgroup of closely related species within the *Bacillus* genus, which may be restricted to the *Bacillus cereus* group.

### 3.3. Specific Response to NO Stress

**Figure 5:**
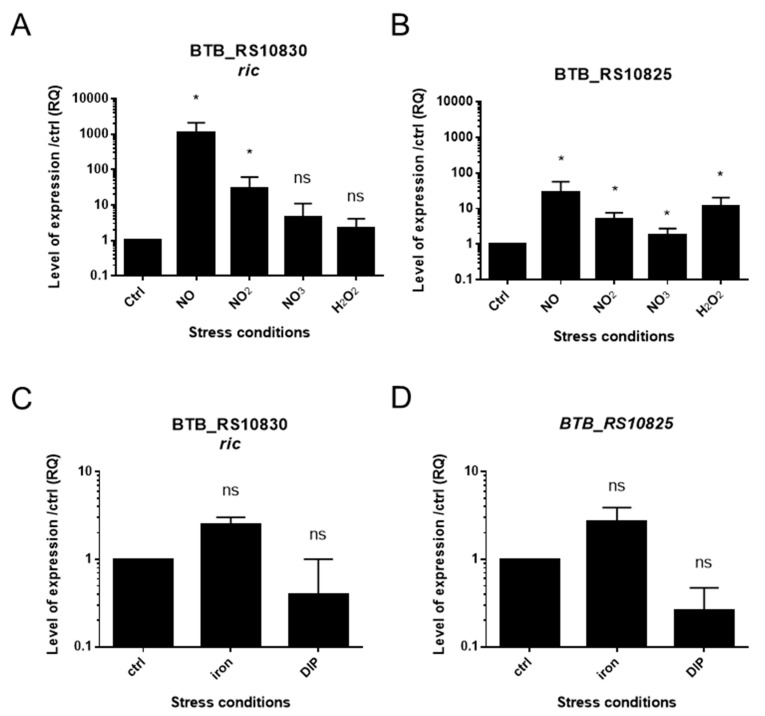
Gene expression analysis by RT-qPCR during different stresses. Gene expression of *ric* and BTB_RS10825 which encodes the large subunit of the nitrite reductase under each condition was normalized to the reference gene *rpoA* and to the non-stressed condition (Ctrl) (RQ: relative quantification). (**A**,**B**) Bacteria were stressed for 15 min with 10 µM of NO, 2.5 mM of sodium nitrite, 20 mM potassium nitrate, 0.03% of hydrogen peroxide, respectively. (**C**,**D**) Bacteria were grown for 1 h in an iron-free medium (RPMI). They were then incubated for 15 min in the presence of excess iron (81 µM of iron citrate) or an iron-specific chelating agent (4,4 μM 2,2′-dipyridyl (DIP)) or RPMI medium alone. Average and standard deviation from three independent experiments are shown. Statistically significant increases of expression in comparison to the control are indicated with asterisks (Mann-Whitney; ns, not significant, * *p*-value < 0.05).

The putative NsrR and FNR regulators binding region were identified by sequence analysis. These regulators have been shown to be involved in resistance to NO and in nitrate/nitrite reductase systems in other species. The impact of NO, nitrite, nitrate on *ric* gene expression and its region was assessed by RT-qPCR (Figure 5).

RT-qPCR analysis confirmed the transcriptional up-regulation of *ric* and BTB_RS10825 (nitrite reductase large subunit) genes in NO (10 μM) conditions, in line with the RNAseq analysis. In addition, a significant up-regulation of the *ric* gene by nitrites was also identified, but not by nitrates. By contrast, the BTB_RS10825 gene encoding the large subunit of nitrite reductase was significantly up-regulated by nitrite and nitrate. To assess whether other oxidizing agents such as H_2_O_2_ also regulates the genes of this region, the bacteria were stressed for 15 min by H_2_O_2_ and gene expression was measured by RT-qPCR. The gene of the nitrite reductase (BTB_RS10825) is up-regulated by hydrogen peroxide, but an increase was not significant for *ric* expression. The expression of *cobA*, BTB_RS10810 and BTB_RS10805 was also investigated, but their basal level of expression was low and close to the noise, so the results were not conclusive (data not shown). As a putative, Fur box was also found in the promoter region of *ric*, the gene expression of the region of interest was investigated to examine a possible impact of iron. The expression of the *ric* and BTB_RS10825 genes might be slightly activated by an excess of iron and inhibited by a starvation, but this observed trend was not statistically significant in pairwise comparisons to the reference.

## 4. Discussion

*Bacillus cereus* is an emerging pathogen capable of resisting macrophages [41]. During phagocytosis, nitric oxide is secreted. Few studies have evaluated the NO concentration in vivo, but it has been estimated that NO could reach 10 µM in the gut during inflammation [42]. To survive this host immune response, pathogenic bacteria have developed means to resist the damages caused by the NO stress. In this study, we highlight a new mechanism by which *B. cereus* responds to NO stress. We show that *B. cereus* up-regulates a large number of genes in response to NO stress, and especially in an early phase of the response. In particular, we identify the up-regulation of six genes linked by their expression pattern and close proximity on the chromosome. This organization appears specific to the *B. cereus* group and may indicate an important mechanism to cope with NO stress.

The mRNA sequencing shows a NO dose and time-dependent effect of the NO stress on gene expression; the early response being more important than the late response. The weaker late response, with less gene transcription modification, suggests an overall resilience, in particular at low concentration. This may be explained by the early and transient up-regulation of genes encoding NO detoxification enzymes such as flavohemoglobin, nitrite reductase, hydroxylamine reductase, which may lead to a decrease in NO concentration afterwards.

During the early response, the most up-regulated genes belong mainly to the siroheme pathway and iron-sulfur cluster repair proteins. In particular, the most up-regulated gene was *ric* (repair of iron centers), which encodes a protein involved in the repair of iron-sulphur clusters of proteins ([Fe-S]). [Fe-S] is an atom group containing two to four iron atoms, linked to S^2−^ sulfide anions present in some proteins [43]. These groups are present on metalloproteins and give them oxido-reduction properties. [Fe-S]-containing proteins are one of the main targets of NO. In turn, some of the [Fe-S] proteins are regulatory proteins whose [Fe-S] react with NO and control their transcriptional or translational activity [44]. NO, as well as its derivatives, thereby modulate the function of these proteins by nitrosylating or disassembling [Fe-S] clusters, which contributes to the effect that NO has on bacteria [45].

The RIC protein is well conserved in prokaryotes and is present in Gram-positive and Gram-negative pathogenic bacteria. The *B. cereus* RIC shows 36.2% identity with its homologue ScdA of *Staphylococcus aureus* (E-value = 2.00E-52), 36% identity with YtfE of *Escherichia coli* (E-value = 4E-40), and 35.7% identity with DnrN or NorA of *Klebsiella pneumoniae* (E-value = 2E-40). YtfE and ScdA possess two atoms of iron. They belong to the RIC family and are involved in the assembly and repair of iron-sulphur groups. The expression of their genes is regulated by FNR and by iron trough Fur. In addition, *ytfE* is regulated by NsrR in *E. coli* but this regulator is absent in *S. aureus* and *B. cereus* [46]. The *scdA* and *ytfE* mutants of *S. aureus* and *E. coli*, are more sensitive to NO than wild-type strains [3,4]. Here we show that *B. cereus ric* expression is up-regulated by NO and nitrite. The sequence analysis highlights putative Fur and NsrR binding sites that might regulate *ric* expression or the entire region. However, the involvement of Fur as a regulator of iron uptake could not be confirmed by our experiments.

The region located between positions 2,097,000 and 2,140,000 of the NC_018877.1 sequence in the neighborhood of *ric* contains five genes downstream and co-directionally transcribed, which are also up-regulated in NO condition. According to NCBI genome annotation these genes are BTB_RS10825 and BTB_RS10820 (*nirD*), that encode the large and small subunits of NADPH-nitrite reductase, and BTB_RS10815, BTB_RS10810 and BTB_RS10805, which encode uroporphyrin-III C-methyltransferase, sirohydrochlorin chelatase and precorrin-2 dehydrogenase, respectively.

NADH-dependent nitrite reductases are present in fungi and bacteria and are used for the assimilation of nitrogen from nitrate or nitrite in anaerobic condition. They are also involved in NO metabolism and resistance. This cytosolic enzyme is an oxidoreductase that catalyzes the reduction of nitrite to ammonia through an [Fe-S] cluster and three cofactors: FAD, iron and siroheme. In *E. coli*, its expression is regulated by FNR [47,48] which is a global regulator that controls numerous pathways, including nitrate and nitrite uptake. ROS and NO resistance and iron might also play a role in FNR regulation [8,49]. In this study, we showed that BTB_RS10825 encoding the *B. cereus* nitrite reductase is up-regulated by NO, nitrate, nitrite and H_2_O_2_, and a FNR binding site was found upstream of its gene.

The three following genes encode three enzymes which are part of the pathway and biosynthesis of siroheme from uroporphyrinogen III [50]. Siroheme is a tetrapyrroleic group that can bind to certain enzymes that have redox activities, including nitrite reductase. This cofactor is used by prokaryotes, plants and fungi, but is absent from higher eukaryotes. It participates in the assimilation of nitrites and sulfites as sources of nitrogen and sulfur [51]. In *Aspergillus fumigatus*, a mutant that does not produce siroheme has diminished resistance to NO and virulence in insects [51].

**Figure 6:**
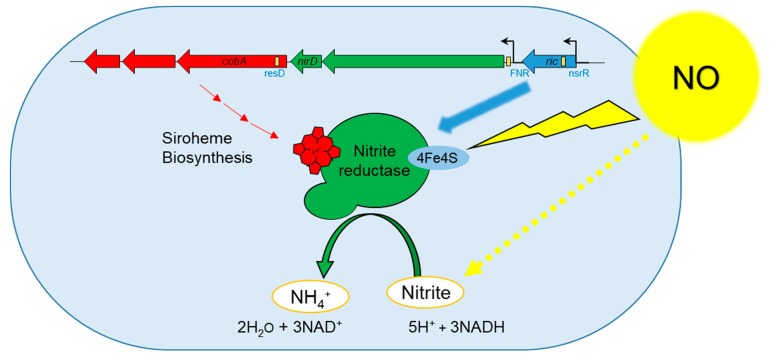
Model of *Bacillus cereus* response to NO. NO can be oxidized into nitrite and the nitrate reductase reduces this still toxic compound to ammonium. The green genes encode the two subunits of the nitrite reductase, which possesses an iron-sulphur cluster and requires a siroheme cofactor. The nitrate reductase genes are co-localized and co-expressed with, on one side, the three genes in red which encode proteins involved in the biosynthesis of siroheme, and on the other side, the *ric* gene in blue, which encodes a protein involved in iron cluster repair.

Taken together, our results point to a new region in the chromosome of *B. cereus* which may play an important role during NO stress. Firstly, the genes in the region are up-regulated in NO conditions. Secondly, the region includes a key enzyme for the metabolism of the bacterium, the nitrite reductase. This enzyme has been previously shown to require a [Fe-S] cluster and siroheme to be functional [52]. These two groups contain at least one iron atom, and are thus targets of NO. Thirdly, the nitrite reductase genes are surrounded by genes involved in the synthesis or repair of these two ferrous moieties. Therefore, *B. cereus* has at its disposal in this single region important ingredients to fight against the host NO response: we can hypothesize that, on one hand, the *ric* gene may repair the sulfur iron cluster of the nitrate reductase, and, on the other hand, the three enzymes encoded by BTB_RS10815, BTB_RS10810 and BTB_RS10805 synthesize siroheme to maintain its reducing activity (Figure 6).

## Figures and Tables

**Table 1:** Consensus sequences of DNA binding sites.

Regulators	Consensus Sequences	Sources
FNR	TGTGANNNNNNTCACA	[37]
NsrR	AANATGCATTT	[38]
ResD	TNTNANNANTNTNTGACAANT	[39]
Fur	GATAATGATAATCATTCT	[40]

**Table 2:** List of qPCR primers used in this study.

Locus Tag	Primer Forward	Primer Reverse
BTB_RS10830	GAAAATGAACATAACCACGCTG	TGTAAACAAGTCGATAAGTGCC
BTB_RS10825	GACCAAATACGCAAATAGCAAG	TTCATAAAGAGGGGCAACAAG
BTB_RS10820	TCATAAAAACGGACCATTAGCC	TGTCACCATCAATGACTTCTAC
BTB_RS10815	CGACAAGCAGGAAAAAAAGAAG	GTAATGAAACGACATCACCAAC
BTB_RS10805	AGAATCATCGTTTCACACACC	GTTCCTGCTTCATACGCTTC
BTB_RS00795	TAACTCCTTACGTCGTATTC	ATTTCCAACGTCTTCTCTTC

## Data Availability

Raw transcriptomic data and differential expression analysis are accessible through GEO Series accession number GSE168681 (https://www.ncbi.nlm.nih.gov/geo/query/acc.cgi?acc=GSE168681, accessed on 1 June 2021).

## Supplementary Materials

The following are available online at https://www.mdpi.com/article/10.3390/ijms22105079/s1, Table S1: RNA sample description; Table S2: List of genes influenced after 15 min of 10 µM NO stress.
