# Supplementary material for: Implication of a Key Region of Six Bacillus cereus Genes Involved in Siroheme Synthesis, Nitrite Reductase Production and Iron Cluster Repair in the Bacterial Response to Nitric Oxide Stress
Source: Int J Mol Sci. 2021 May 11;22(10):5079. doi: 10.3390/ijms22105079 (PMC8151001; doi:10.3390/ijms22105079)
Supplement: Supplementary file 1 [file ijms-22-05079-s001.zip › ijms-1166125-supplementary.pdf]

Suppl 1: RNA sample description

Sample name	CFU/mL	Sample description	Griess assay [NO] (µM)	RIN Bioanalyseur	volume (µl)	concentration QuBit(ng/µl)	Total reads
R12-To	1,05E+06	Initial condition: T = 0 of replicate n ° 12	1,3	8,9	10	152	19 032 476
R12-15A	2,75E+06	Control condition (without stress) after 15min at 37°	1,3	9,3	10	141	22 683 579
R12-15B	3,40E+06	15 min incubation with 10µM of NO	10,5	10	10	220	18 884 247
R12-15C	1,40E+06	15 min incubation with 50µM of NO	64,5	9,2	10	147	19 975 417
R12-1A	4,95E+06	Control condition (without stress) after 60min at 37°	1,8	10	10	116	20 970 283
R12-1B	5,40E+06	60 min incubation with 10µM of NO	6,1	9,8	10	138	20 242 855
R12-1C	3,55E+06	60 min incubation with 50µM of NO	38,9	9,8	10	121	18 736 994
R14-To	1,08E+06	Initial condition: T = 0 of replicate n ° 14	1,8	9,6	10	118	20 124 783
R14-15A	8,65E+05	Control condition (without stress) after 15min at 37°	2,1	9,4	10	114	20 791 322
R14-15B	1,16E+06	15 min incubation with 10µM of NO	9,1	9,8	10	108	21 988 766
R14-15C	9,05E+05	15 min incubation with 50µM of NO	60,8	9,9	10	107	27 184 430
R14-1A	1,79E+06	Control condition (without stress) after 60min at 37°	2,9	9,7	10	109	21 530 597
R14-1B	1,52E+06	60 min incubation with 10µM of NO	3,0	9,9	10	103	20 954 601
R14-1C	1,85E+06	60 min incubation with 50µM of NO	44,5	10	10	95,2	22 312 588
R16-To	9,00E+05	Initial condition: T = 0 of replicate n ° 16	0,2	10	10	110	21 238 399
R16-15A	1,50E+06	Control condition (without stress) after 15min at 37°	0,3	9,9	10	144	19 801 942
R16-15B	3,05E+06	15 min incubation with 10µM of NO	8,5	9,9	10	111	20 500 137
R16-15C	1,98E+06	15 min incubation with 50µM of NO	57,2	9,8	10	130	20 124 601
R16-1A	5,25E+06	Control condition (without stress) after 60min at 37°	0,1	9,3	10	139	19 657 553
R16-1B	5,25E+06	60 min incubation with 10µM of NO	4,7	9,9	10	110	19 306 037
R16-1C	3,90E+06	60 min incubation with 50µM of NO	33,7	9,4	10	146	19 275 840

Suppl 2: List of genes influenced after 15 min of 10 µM NO stress.

"All effect code" reflects the activation (U) or inhibition (D) of gene transcription compared with the control condition without NO. The position in the chromosome is indicated as well as the protein identification number in NCBI.

locus_tag	Start	End	gene	Effect (at 15min, 10µM vs 0µM)	qvalue	all effects code	product	protein_id
BTB_RS10830	2102904	2103611	*ric*	8,7301	0	UU-U	iron-sulfur cluster repair di-iron protein	WP_000679632,1
BTB_RS07355	1434746	1435954		5,7842	0	UU-U	NO-inducible flavohemoprotein	WP_000947339,1
BTB_RS17205	3399147	3400433		5,3262	0	UU--	hydroxylamine reductase	WP_002094103,1
BTB_RS09885	1925663	1927387	*cydC*	4,2864	0	UU-U	thiol reductant ABC exporter subunit CydC	WP_000073638,1
BTB_RS10820	2099957	2100274	*nirD*	4,23	0,0088	UU--	nitrite reductase small subunit NirD	WP_000616683,1
BTB_RS10810	2097767	2098477		4,2279	0	UU--	sirohydrochlorin chelatase	WP_000676477,1
BTB_RS09880	1923945	1925666	*cydD*	3,9212	0	UU-U	thiol reductant ABC exporter subunit CydD	WP_000824029,1
BTB_RS09875	1922929	1923945	*cydB*	3,6701	0	UU-U	cytochrome d ubiquinol oxidase subunit II	WP_000950083,1
BTB_RS04380	851653	851931		3,5831	0,0075	UU--	hypothetical protein	WP_000900043,1
BTB_RS10815	2098474	2099898	*cobA*	3,4912	0,0008	UU--	uroporphyrin-III C-methyltransferase	WP_001014975,1
BTB_RS10805	2097301	2097774		3,1919	0,0032	UU--	precorrin-2 dehydrogenase	WP_000282601,1
BTB_RS18075	3592867	3594723	*nrdD*	3,1698	0,0003	UU--	anaerobic ribonucleoside triphosphate reductase	WP_000940080,1
BTB_RS10825	2100290	2102695		3,1545	0,0015	UU--	NADPH-nitrite reductase large subunit	WP_000746957,1
BTB_RS09870	1921539	1922942		3,0694	0	UU-U	cytochrome ubiquinol oxidase subunit I	WP_000448869,1
BTB_RS25855	5049165	5050859		3,0186	0,0008	UU--	methyl-accepting chemotaxis protein	WP_000878450,1
BTB_RS05820	1148439	1149104		2,9268	0,0044	U---	S-layer homology domain-containing protein	WP_011109902,1
BTB_RS26535	5187990	5189315		2,8801	0,0095	UU-U	6-phospho-beta-glucosidase	WP_000145762,1
BTB_RS04385	851921	853006		2,8741	0,0002	UU--	DUF871 domain-containing protein	WP_000440050,1
BTB_RS22185	4366170	4368773		2,6752	0,0339	UU--	bifunctional acetaldehyde-CoA/alcohol dehydrogenase	WP_000260464,1
BTB_RS04375	850367	851677		2,6714	0,01	UU--	PTS sugar transporter subunit IIC	WP_001013042,1
BTB_RS03680	731060	731458		2,613	0,0136	UU--	DUF3221 domain-containing protein	WP_000494384,1
BTB_RS18840	3740505	3742424		2,6037	0,0098	U---	LTA synthase family protein	WP_000791042,1
BTB_RS02515	498240	498971	*pflA*	2,5627	0,0089	UU--	pyruvate formate lyase-activating protein	WP_000238463,1
BTB_RS23470	4624070	4624528		2,5521	0,0259	UU--	universal stress protein	WP_001066673,1
BTB_RS10425	2029474	2030121		2,5263	0,008	UU--	CBS domain-containing protein	WP_001220528,1
BTB_RS05410	1062076	1063758		2,4369	0,0026	UU-U	aerobic glycerol-3-phosphate dehydrogenase	WP_000672829,1
BTB_RS26540	5189319	5189642		2,3819	0,0295	UU--	PTS lactose/cellobiose transporter subunit IIA	WP_000989057,1
BTB_RS02095	401194	401643	*argR*	2,3409	0,0162	UU--	arginine regulator	WP_000711826,1
BTB_RS04370	849978	850283		2,322	0,0163	UU--	PTS sugar transporter subunit IIB	WP_000275645,1
BTB_RS21135	4181630	4182949		2,3043	0,0158	UU--	2-oxo acid dehydrogenase subunit E2	WP_000257645,1
BTB_RS14280	2824130	2825497		2,2156	0,0056	UU--	chitin-binding protein	WP_001065156,1
BTB_RS34095	5078483	5078575		2,2088	0,0101	U--U	hypothetical protein	WP_078994172,1
BTB_RS05340	1049356	1050333		2,2064	0,0077	UU--	ring-cleaving dioxygenase	WP_001072559,1
BTB_RS04355	846440	847528		2,193	0,0003	UU--	DUF871 domain-containing protein	WP_001253350,1
BTB_RS04365	849656	849976		2,1803	0,0236	UU--	PTS lactose/cellobiose transporter subunit IIA	WP_000774624,1
BTB_RS09865	1919891	1920946		2,0865	0,0197	UU--	LLM class flavin-dependent oxidoreductase	WP_000417549,1
BTB_RS02510	495921	498170	*pflB*	2,0756	0,0072	UU--	formate C-acetyltransferase	WP_000195468,1
BTB_RS26175	5116350	5117645		2,073	0,0029	UU--	enolase	WP_000103951,1
BTB_RS10485	2040295	2041263		2,0441	0,0344	UU-D	SPFH/Band 7/PHB domain protein	WP_000226257,1
BTB_RS26195	5121314	5122318	*gap*	2,0255	0,0007	UU--	aldehyde dehydrogenase	WP_000161234,1
BTB_RS21555	4248583	4249278		2,0147	0,0154	UU--	transcriptional regulator	WP_000434124,1
BTB_RS21150	4184991	4186412	*lpdA*	2,0122	0,0386	UU--	dihydrolipoyl dehydrogenase	WP_001051295,1
BTB_RS11230	2188967	2189554		1,9938	0,0039	UU--	SCO family protein	WP_000833332,1
BTB_RS03420	691216	692268		1,993	0,0087	UU--	(RR)-butanediol dehydrogenase	WP_000645827,1
BTB_RS21140	4182965	4183948		1,9755	0,0282	UU--	3-methyl-2-oxobutanoate dehydrogenase subunit beta	WP_000290071,1
BTB_RS18820	3734300	3735289		1,9648	0,0095	UU--	1-phosphatidylinositol phosphodiesterase	WP_000066296,1
BTB_RS11320	2204796	2205833		1,94	0,0211	UU--	alcohol dehydrogenase AdhP	WP_000649129,1
BTB_RS18070	3592418	3592870	*nrdG*	1,9298	0,0156	UU--	anaerobic ribonucleoside-triphosphate reductase activating protein	WP_000867078,1
BTB_RS05225	1024524	1024964		1,9227	0,0087	UU--	bacterioferritin	WP_000017752,1
BTB_RS08240	1609064	1609897		1,9096	0,0491	UU-D	flagellar motor protein MotP	WP_000504188,1
BTB_RS21155	4186417	4187520		1,9005	0,0485	UU-D	butyrate kinase	WP_000115781,1
BTB_RS14995	2947186	2947833		1,893	0,0487	UU--	DJ-1/PfpI family protein	WP_000407026,1
BTB_RS23670	4660527	4661171		1,8841	0,0078	UU---	CBS domain-containing protein	WP_000634583,1
BTB_RS26180	5117676	5119205		1,882	0,0076	UU--	23-bisphosphoglycerate-independent phosphoglycerate mutase	WP_001231158,1
BTB_RS33630	3643789	3643938		1,881	0,0289	UU--	hypothetical protein	WP_002152557,1
BTB_RS02770	535206	535856		1,8806	0,0002	UU--	DsbA family protein	WP_000841308,1
BTB_RS11170	2176905	2178362		1,8684	0,0381	UU-U	serine hydrolase	WP_000034317,1
BTB_RS21145	4183962	4184963		1,8678	0,0461	UU--	3-methyl-2-oxobutanoate dehydrogenase subunit alpha	WP_000852551,1
BTB_RS26135	5107957	5108724		1,8587	0,0366	UU--	N-acetyltransferase	WP_000632238,1
BTB_RS23675	4661168	4662334		1,8572	0,0328	UU--	acetoin utilization protein AcuC	WP_000092829,1
BTB_RS03095	618068	619687		1,8533	0,0309	UU--	L-lactate permease	WP_000109099,1
BTB_RS18345	3643065	3643772		1,8525	0	UU-U	cytochrome c biogenesis protein CcdA	WP_001152676,1
BTB_RS07195	1403404	1404375		1,8433	0,0359	UU--	2-hydroxyacid dehydrogenase	WP_001062841,1
BTB_RS26185	5119202	5119957		1,8429	0,0127	UU--	triose-phosphate isomerase	WP_001231038,1
BTB_RS25665	5012914	5013864		1,8354	0,0328	UU--	L-lactate dehydrogenase	WP_000820660,1
BTB_RS03825	760551	760862	*qoxD*	1,7956	0,0009	UU--	cytochrome aa3 quinol oxidase subunit IV	WP_000068425,1
BTB_RS04360	847605	849545		1,7384	0,0009	UU--	transcription antiterminator	WP_000169804,1
BTB_RS26130	5106784	5107461		1,7376	0,0137	UU--	response regulator transcription factor	WP_000815803,1
BTB_RS09940	1937282	1937881		1,7357	0,0426	UU--	nitroreductase family protein	WP_001029924,1

BTB_RS05335	1048356	1049300		1,7261	0,046	UU--	ring-cleaving dioxygenase	WP_000272975,1
BTB_RS32215	4686	5030		1,6936	0,0352	UU-D	DUF3139 domain-containing protein	WP_000968001,1
BTB_RS01255	222548	222667		1,688	0,0234	UU--	DUF3948 family protein	WP_003269239,1
BTB_RS02310	453260	455926		1,6684	0,0483	UU--	cation-translocating P-type ATPase	WP_000073697,1
BTB_RS08185	1601714	1602253		1,6606	0,0085	UU--	sigma-70 family RNA polymerase sigma factor	WP_000575582,1
BTB_RS32210	3827	4639		1,6409	0,0276	UU--	hypothetical protein	WP_000734781,1
BTB_RS26190	5119990	5121174	*pgk*	1,6245	0,0374	UU--	phosphoglycerate kinase	WP_001036331,1
BTB_RS03830	760863	761465	*qoxC*	1,6152	0,0032	UU--	cytochrome aa3 quinol oxidase subunit III	WP_000729314,1
BTB_RS23465	4623131	4623922	*fabG*	1,6106	0,0167	UU--	SDR family oxidoreductase	WP_002024902,1
BTB_RS06615	1303659	1305071	*glcD*	1,5991	0,0157	UU--	glycolate oxidase subunit GlcD	WP_000890756,1
BTB_RS11120	2169335	2169775		1,5879	0,0066	UU-D	GNAT family N-acetyltransferase	WP_001223866,1
BTB_RS26650	5212876	5214537		1,5765	0,0289	UU--	L-lactate permease	WP_001099343,1
BTB_RS18380	3649868	3650770		1,567	0,0216	UU--	LysR family transcriptional regulator	WP_000423235,1
BTB_RS03015	597923	599056	*ald*	1,5549	0,0383	UU--	alanine dehydrogenase	WP_001219741,1
BTB_RS03020	599160	600575		1,5415	0,0334	UU--	amino acid permease	WP_001284897,1
BTB_RS05625	1106047	1107468		1,5182	0,0302	UU--	protoporphyrinogen oxidase	WP_001228113,1
BTB_RS04285	833443	835107	*cdr*	1,507	0,0483	UU--	CoA-disulfide reductase	WP_000087589,1
BTB_RS25975	5074011	5078432		1,5049	0,0118	UU--	wall-associated protein precursor	WP_000038391,1
BTB_RS04160	812092	812946		1,4972	0,0246	UU-D	patatin family protein	WP_000892676,1
BTB_RS11600	2261170	2261369		1,4949	0,0376	UU--	hypothetical protein	
BTB_RS03835	761479	763422	*qoxB*	1,4753	0,0031	UU--	cytochrome aa3 quinol oxidase subunit I	WP_003269518,1
BTB_RS04525	876775	878139		1,4208	0,0428	UU--	permease	WP_000711293,1
BTB_RS08190	1602246	1602866		1,4092	0,0143	UU--	flagellar motor switch protein FliG	WP_078994751,1
BTB_RS11965	2340828	2342219		1,36	0,0165	UU--	protoporphyrinogen oxidase	WP_000860354,1
BTB_RS17195	3397922	3398263		1,347	0,0428	UU--	DUF1048 domain-containing protein	WP_000891986,1
BTB_RS01250	222397	222513		1,334	0,0481	UU--	DUF3948 family protein	WP_000654250,1
BTB_RS25875	5055059	5055757		1,3248	0,0225	UU--	pirin family protein	WP_000488929,1
BTB_RS22635	4460551	4461480		1,3209	0,0352	UU--	hydroxymethylbilane synthase	WP_001226397,1
BTB_RS04590	886427	887629		1,249	0,0472	UU-D	tetracycline resistance MFS efflux pump	WP_000742960,1
BTB_RS01960	372643	374169		1,2352	0,0204	UU--	alkyl hydroperoxide reductase subunit F	WP_000599497,1
BTB_RS26435	5164106	5164429		1,2069	0,0054	UU--	cytochrome c-551	WP_000727975,1
BTB_RS18000	3576893	3577930		1,1993	0,0409	UU--	LytR family transcriptional regulator	WP_000427598,1
BTB_RS22645	4462347	4463681		1,1972	0,0286	UU--	glutamyl-tRNA reductase	WP_000547870,1
BTB_RS23665	4659877	4660509		1,184	0,0487	UU--	acetoin utilization protein acetyltransferase AcuA	WP_000581393,1
BTB_RS08310	1623295	1623663		1,1296	0,0263	UU--	flagellar biosynthesis protein FliS	WP_001149516,1
BTB_RS22630	4459796	4460548		1,0259	0,0138	UU-u	uroporphyrinogen-III synthase	WP_000992351,1
BTB_RS08315	1623647	1623925		0,9972	0,033	uU--	flagellar biosynthesis protein FlgG	WP_074554103,1
BTB_RS16655	3288832	3289851		0,952	0,0348	u---	tyrosine-protein phosphatase	WP_000437806,1
BTB_RS08385	1634818	1635726		0,908	0,039	uU--	chemotaxis signal transduction protein CheV	WP_000075943,1
BTB_RS24905	4880922	4882112		0,873	0,019	uu--	aminotransferase	WP_000808531,1
BTB_RS10125	1975377	1976819		-0,8483	0,0469	dD--	PLP-dependent aminotransferase family protein	WP_000439423.1
BTB_RS11195	2183152	2183871		-1,0024	0,0122	DD-D	1-acyl-sn-glycerol-3-phosphate acyltransferase	WP_000616582.1
BTB_RS00635	121371	121793		-1,0484	0,0315	DD--	30S ribosomal protein S12	WP_001142341.1
BTB_RS06810	1342164	1342610		-1,1618	0,0105	DD--	MarR family transcriptional regulator	WP_000203336.1
BTB_RS06835	1346920	1347582		-1,1885	0,0162	DD-U	7-cyano-7-deazaguanine synthase QueC	WP_000711595.1
BTB_RS24210	4759529	4759852		-1,2019	0,0338	DD-d	iron-sulfur cluster biosynthesis family protein	WP_000289570.1
BTB_RS13815	2733031	2733411		-1,2984	0,0141	DD--	nucleotide excision repair endonuclease	WP_001048947.1
BTB_RS00820	143574	144317		-1,3273	0,0371	DD--	tRNA pseudouridine(38-40) synthase TruA	WP_015055073.1
BTB_RS00615	112821	113420		-1,3536	0,0308	DD--	class I SAM-dependent methyltransferase	WP_000763269.1
BTB_RS20705	4103724	4104797		-1,3738	0,038	DD--	ABC transporter permease	WP_074618732.1
BTB_RS07040	1377055	1377846		-1,4064	0,0096	DD-D	bacitracin resistance undecaprenyl-diphosphatase	WP_000796253.1
BTB_RS06410	1262883	1263293		-1,4221	0,0126	DD-d	DUF3908 domain-containing protein	WP_000996808.1
BTB_RS27390	5374183	5375220		-1,4888	0,0014	DD-D	transcriptional regulator	WP_000996313.1
BTB_RS17935	3559280	3560614		-1,5549	0,0092	DD-D	amino acid permease	WP_078400513.1
BTB_RS26580	5195686	5196903		-1,8217	0,029	DD-D	dicarboxylate/amino acid:cation symporter	WP_000649376.1
BTB_RS03775	748968	750269		-1,9306	0,0002	DD-D	branched-chain amino acid transport system II carrier protein	WP_000771703.1
BTB_RS05575	1089279	1095841		-2,011	0,0493	DD-U	adhesin	
BTB_RS10715	2075927	2076136		-2,0689	0,0253	DD--	DUF3925 domain-containing protein	WP_000844984.1
BTB_RS17755	3519702	3520322		-2,075	0,0076	DD--	transcriptional regulator	WP_000070938.1
BTB_RS33410	2999506	2999712		-2,7749	0,0406	DD--	hypothetical protein	
